# Investigating balance, gait, and physical function in people who have undergone thoracic surgery for a diagnosis of lung cancer: A mixed-methods study

**DOI:** 10.1177/14799731211052299

**Published:** 2021-10-29

**Authors:** Daniel Tough, Joel Dunning, Jonathan Robinson, John Dixon, Jonathan Ferguson, Ian Paul, Samantha L Harrison

**Affiliations:** 1 School of Health and Life Sciences, 5462 Teesside University, Middlesbrough, UK; 2 Department of Cardiothoracic Surgery, 156705 James Cook University Hospital, Middlesbrough, UK

**Keywords:** Oncology, cancer, lung cancer, falls, balance, gait

## Abstract

**Objectives:**

Symptoms associated with lung cancer and thoracic surgery might increase fall risk. We aimed to investigate: 1) balance, gait and functional status in people post-thoracic surgery compared to healthy controls; 2) perceptions of balance, gait and functional status.

**Methods:**

Recruitment targeted older adults (≥50 years) who had undergone thoracic surgery for a diagnosis of lung cancer in the previous 3 months, and healthy age-matched controls. Dynamic and static balance, gait velocity, knee-extension strength and physical activity levels were assessed using the BESTest, Kistler force plate, GAITRite system, Biodex System 3 and CHAMPS questionnaire, respectively. Two-part semi-structured interviews were conducted post-surgery.

**Results:**

Individuals post-surgery (*n* = 15) had worse dynamic balance and gait, and lower levels of moderate/vigorous physical activity (MVPA) (all *p*<0.05) versus healthy controls (*n* = 15). Strength did not differ between groups (*p* > 0.05). No associations between BESTest and strength or physical activity existed post-surgery (*p* > 0.05). Three themes were identified: 1) Symptoms affect daily activities; 2) Functional assessments alter perceptions of balance ability and 3) Open to supervised rehabilitation.

**Conclusion:**

Balance, gait and MVPA are impaired post-thoracic surgery, yet balance was not viewed to be important in enabling activities of daily living. However, supervised rehabilitation was considered acceptable.

## Introduction

Lung cancer is the leading cause of cancer death.^1^ Whilst thoracic surgery is the most effective lung cancer treatment, it is associated with pain, quadriceps and respiratory muscle weakness, dyspnoea, cancer-related fatigue, phrenic nerve damage, and an altered centre of gravity (COG).^2–7^ Some symptoms, such as pain, will improve with time; however, others (e.g. altered COG and dyspnoea) may be longer lasting. These symptoms have been associated with balance and gait impairments in older adults,^8,9^ potentially increasing the risk of falling post-surgery, although risk factors for falls have not yet been examined in this population. These symptoms may also be experienced due to postural changes attributed to chronic respiratory disease prior to surgery.^10^ One cross-sectional study reported an increase in incidence of falls in people with lung cancer one to 2 years post-diagnosis (28%) compared to pre-diagnosis (17%), greater than the increase seen in other cancer types, including breast, colorectal and prostate.^9^ Another study showed the impact that cancer treatment, including chemotherapy, has on increasing falls incidence, with 25% of people falling who had cancer but received no treatment versus 33% of people who did receive treatment.^11^

Understanding individuals’ views about their balance, gait and broader functional status is important to inform the content and delivery of an acceptable intervention tailored to meet their specific needs. Qualitative research is often used to glean this type of information,^12,13^ yet it has seldom been applied in surgical settings.^14^

The primary aims of this study are to: 1) assess whether risk factors for falls (balance and gait) and functional status are impaired in people post-thoracic surgery for a diagnosis of lung cancer compared to healthy adults of the same age; and 2) investigate the perceptions of individuals with lung cancer on their balance and gait. Secondary aims are: 1) To investigate associations between balance and: a) quadriceps strength; and b) physical activity (PA) levels; and 2) To gain an insight into individuals’ preferences for the content and delivery of rehabilitation post-thoracic surgery.

## Methods

### A study design

An observational mixed-method study design was applied. Quantitative data were collected to assess differences in risk factors for falls and functional status between people with lung cancer and healthy individuals. Qualitative data allowed the exploration of participants’ opinions on balance, gait and functional status post-surgery. Ethical approval was obtained from Teesside University’s School of Health and Social Care research ethics committee (REC) (125/17), the Health Research Authority, Leicester South REC (18/EM/0115) and South Tees Hospitals NHS Foundation Trust research and development department (2018035). All participants provided informed consent.

### A participants and recruitment

Individuals post-thoracic surgery for lung cancer were recruited from the cardiothoracic surgery department at a local hospital between December 2018 and March 2020. Healthy age-matched controls were recruited by approaching patients’ family or friends who attended the cardiothoracic clinics. A sample size estimation was carried out using the Balance Evaluation Systems Test (BESTest) from a previous study in individuals with chronic obstructive pulmonary disease (COPD),^15^ with an alpha value of 0.05 and a power value of 0.9. It revealed a sample size of 34 (17 in each group) to detect a change of 13–17 points, which has been quoted to be the minimal clinically important difference (MCID) in a respiratory population.^16^

Individuals with lung cancer were deemed eligible if they: 1) were ≥50 years at the time of the study visit; 2) had the ability to understand English and provide written informed consent; 3) had undergone thoracic surgery for a primary diagnosis of lung cancer in the past 3 months. Anyone who was undergoing or had scheduled adjuvant therapy prior to the study visit was excluded. Healthy controls were eligible if they considered themselves to be healthy (free from chronic conditions) and aged ≥50 years.

Individuals currently with, or had a history of, a cognitive impairment, thus unable to fully understand the study and/or follow assessment instructions, a musculoskeletal or neuromuscular condition which impaired balance and/or mobility (e.g. Parkinson’s disease or multiple sclerosis), or were unable to read and understand English were excluded.

### Data collection

#### Quantitative phase

Anthropometric and demographic data were obtained for all participants. Surgical details were recorded from the thoracic database, including mode of surgery, cancer type and stage. Participants completed a study visit consisting of the following assessments, which were carried out by the lead researcher (DT).

The BESTest^17^ is a comprehensive measure of balance, consisting of 36 items and assesses performance of six balance control systems. Total scores are presented as a percentage (0–100%), with higher scores indicating better balance. It is valid, reliable, and sensitive to change in people with COPD and older adults.^18^

Kistler force plate (Model 9826AA, Kistler Instruments Ltd., Hampshire, UK) assessed centre of pressure (CoP) displacement using stationary bipedal and unipedal stances, with eyes open and a bipedal stance with eyes closed. Each was performed three times for 10 s, with mean values calculated.^19–23^

Platinum GAITRite portable gait analysis system (GAITRitePlatinum, CIR systems Inc, NJ, USA) assessed three five-metre walks, beginning two metres before the start, and ending two metres after the mat.^24,25^ Spatio-temporal outcomes were derived. The mean results of the three walks were calculated.

Biodex System 3 (BIODEX Medical Systems, Shirley, NY, USA) isokinetic dynamometer assessed isometric knee-extension muscle strength. Participants were seated in the Biodex chair at an incline of 90°. Stabilising straps were placed across the participants’ chest, waist and quadriceps.^26,27^ Participants maximally extended their leg against the stationary resistance for 5 seconds, followed by 60 s rest,^28,29^ completing three repetitions on each leg. Mean peak torque normalised by body weight was calculated.

The Community Healthy Activities Model Program for Seniors (CHAMPS)^30^ is a valid and reliable tool to assess PA levels among older populations.^31,32^ It is a 41-item scale which assesses average PA levels within the previous 4 weeks.^33,34^ Frequency and duration participants spent doing mild to vigorous PA and moderate to vigorous PA (MVPA) was calculated.

#### Qualitative phase

A thematic framework informed the semi-structured interview schedule (Supplement 1) which was informed by previous literature (including balance and gait impairment among older adults, symptoms following thoracic surgery (e.g. dyspnoea and pain), and post-surgery rehabilitation programmes), discussions with the surgical team, and individuals post-thoracic surgery (*n* = 7). Questions were amended and added to the framework as they were identified.

DT conducted the interviews in a quiet university laboratory. He is a physiologist by background and was not known to participants. DT also undertook a four-hour qualitative research training course and received mentorship from SH who is experienced in qualitative research methods, including interviewing techniques. The interviews were divided into two parts: 1) prior to any physical assessments examining participants’ perceptions of their balance, gait and functional status post-surgery; and 2) following the physical assessments gleaning their opinion on the content and delivery of a potential rehabilitation intervention offered post-surgery.

### Data analysis

#### Quantitative phase

An independent samples t-test, using Statistical Package for Social Sciences version 23 (SPSS Inc., Chicago, IL), evaluated differences between groups. The mean differences (95% CI) are presented. The alpha value was set a priori at ≤0.05. A Pearson correlation was ran to determine any associations between total BESTest score, as the primary outcome measure, and: 1) quadriceps strength and 2) PA levels.

#### Qualitative phase

All interviews were recorded and transcribed verbatim by a professional transcriber. Data were stored and organised using NVivo (QSR NVivo version 11; QSR International, Doncaster, Australia) and analysed using deductive thematic analysis.^35^

A six-step procedure was followed^35^: 1) Two researchers (DT/JR) independently familiarised themselves with the data of two transcripts, prior to separately generating a list of themes; 2) The researchers met to confirm these themes, prior to organising these into overarching themes; 3) which were then agreed; 4) These were discussed with a third researcher (SH), and; 5) names and definitions were refined. Thematic mapping was used to explore the relationship between themes in the context of the whole dataset; 6) Finally, data extracts were selected by DT to support each theme and written up as a final report. Both interviews (pre- and post-assessment) were analysed simultaneously to enable comparison.

## Results

### Quantitative findings

#### Study population

**Figure 1.**
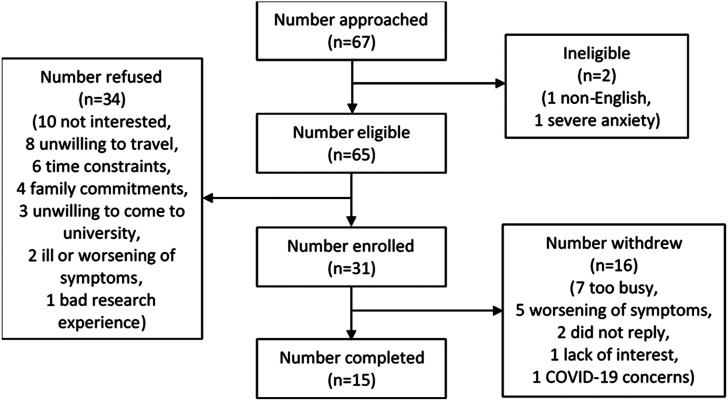
Flow diagram of post-surgery group recruitment.

Sixty-seven individuals with lung cancer were screened, with 31 recruited to the study (Figure 1), and 15 individuals who completed the study. Nine (60%) were male, with a mean (standard deviation (SD)) age of 67 (7) years, a body mass index (BMI) of 28.1 (5.3) and 26 (23) pack years. Thirteen consecutive individuals were invited to partake in the semi-structured interviews, all of whom accepted. Fifteen healthy controls were recruited to the study. Six (40%) were male, with a mean (SD) age of 65 (10) years, a BMI of 25.9 (2.4) and 7 (11) pack years. The only significant between group difference was found in smoking pack years (lung cancer: 26 pack years v controls: 7 years) (*p* = 0.007). Between group differences are displayed in Table 1.

**Table 1.** Physical assessment participant demographics.

<!--Col Count:4-- >	(*n* = 30)		
	Post-surgery (*n* = 15)	Control (*n* = 15)	*p* Value
Age (years)	67 ± 7	65 ± 10	0.411
Sex (*n*) Male Female	9 6	6 9	0.289
BMI	28.1 ± 5.3	25.9 ± 2.4	0.165
Marital status (*n*) Married Divorced Widowed Partner	8 2 5 0	11 1 0 3	0.742
Living arrangement (*n*) House Bungalow Flat	12 2 1	9 3 3	0.217
Lives with (*n*) Spouse Family Alone	8 2 5	10 2 3	0.571
Smoking pack years	26 ± 23	7 ± 11	0.007**
Cancer stage (*n*) Ia Ib IIa IIb IIIa	5 2 4 1 3	—	—
Mode of surgery (*n*) Thoracotomy VATS RATS	5 8 2	—	—
Days between surgery and study visit	46 ± 18	—	—

Data are mean ± SD unless stated. BMI: body mass index; VATS: video-assisted thoracoscopic surgery; RATS: robotic-assisted thoracoscopic surgery.

#### The BESTest

Healthy controls had a higher total BESTest score than people with lung cancer (mean difference (95% CI): 19.9% (11.3 to 29.9%)) and performed better on all sub-scales (*p*<0.05). The greatest between group differences were observed in the sub-scales ‘postural responses’ (22.9% (9.4 to 37.7%)) and ‘biomechanical constraints’ (22.2% (12.2 to 32.3%)) (Table 2).

**Table 2.** Physical assessment results.

<!--Col Count:6-- >	Post-surgery	Control	Mean difference (95% CI)	*p* Value	Effect size (d)
BESTest (%)	(*n* = 15)	(*n* = 15)	—	—	—
Total	68.1 (17.4)	88.0 (7.2)	20.0 (11.3 to 29.9)	**0.003**	1.49
Biomechanical constraints	68.0 (16.0)	90.2 (10.0)	22.2 (12.2 to 32.3)	**0.000**	1.66
Stability limits/verticality	67.6 (19.5)	87.0 (10.7)	19.4 (7.5 to 31.3)	**0.003**	1.23
Anticipatory posture adjustments	67.0 (20.2)	85.9 (9.1)	18.9 (8.3 to 30.0)	**0.007**	1.21
Postural responses	62.6 (25.8)	85.5 (12.0)	22.9 (9.4 to 37.7)	**0.008**	1.14
Sensory orientation	76.0 (17.2)	90.7 (8.6)	14.7 (5.6 to 24.8)	**0.033**	1.08
Stability in gait	68.6 (19.5)	89.5 (8.7)	20.9 (11.2 to 31.6)	**0.004**	1.38
	—	—	—	—	—
CoP displacement	(*n* = 11)	(*n* = 13)	—	—	—
Eyes open AP SD (mm) Range (mm) ML SD (mm) Range (mm) Eyes closed AP SD (mm) Range (mm) ML SD (mm) Range (mm)	5.3 (1.2) 25.0 (6.9) 2.3 (1.2) 11.7 (4.8) 6.6 (3.4) 31.9 (20.7) 2.7 (1.8) 14.5 (9.8)	4.5 (1.2) 20.0 (5.4) 2.1 (0.9) 11.6 (4.7) 4.9 (1.6) 22.5 (6.5) 2.8 (2.2) 11.6 (4.6)	0.7 (−0.3 to 1.7) 5.0 (−0.4 to 10.4) 0.2 (−0.7 to 1.1) 0.2 (−3.9 to 4.2) 1.7 (−0.2 to 3.9) 9.4 (−0.8 to 22.4) 0.1 (−1.8 to 1.6) 2.9 (−4.1 to 9.8)	0.146 0.065 0.599 0.939 0.165 0.225 0.921 0.389	0.60 0.82 0.22 0.03 0.66 0.64 0.04 0.38
	—	—	—	—	—
GAITRite	(*n* = 15)	(*n* = 15)	—	—	—
Step time (s) Left Right	0.56 (0.05) 0.56 (0.06)	0.51 (0.03) 0.51 (0.02)	0.05 (0.03 to 0.08) 0.05 (0.02 to 0.08)	**0.005** **0.023**	1.21 1.12
Cycle time (s) Left Right	1.12 (0.10) 1.12 (0.11)	1.02 (0.05) 1.02 (0.05)	0.10 (0.05 to 0.15) 0.10 (0.05 to 0.15)	**0.011** **0.014**	1.26 1.17
Step length (cm) Left Right	59.3 (10.5) 58.9 (11.0)	62.8 (5.1) 63.1 (5.7)	3.5 (−2.0 to 9.0) 4.2 (−1.7 to 9.9)	0.260 0.203	0.42 0.48
Heel-to-heel base of support (cm) Left Right	12.1 (3.3) 11.8 (3.1)	8.7 (2.6) 8.6 (2.7)	3.5 (1.2 to 5.7) 3.1 (0.9 to 5.3)	**0.004** **0.007**	1.14 1.10
Single support time (%GC) Left Right	36.7 (2.3) 36.9 (2.7)	37.7 (1.7) 38.1 (1.0)	1.0 (−0.5 to 2.6) 1.2 (−0.3 to 2.8)	0.166 0.112	0.49 0.59
Double support time (%GC) Left Right	27.7 (5.0) 27.8 (5.1)	25.0 (3.7) 25.0 (3.6)	2.7 (−0.6 to 6.0) 2.8 (−0.5 to 6.1)	0.108 0.092	0.61 0.63
Swing time (%GC) Left Right	36.4 (2.6) 36.5 (2.2)	38.4 (0.7) 38.0 (1.6)	1.9 (0.8 to 3.3) 1.5 (0.1 to 3.0)	**0.022** **0.035**	1.05 0.78
Stance time (%GC) Left Right	63.6 (2.6) 63.5 (2.2)	61.7 (0.7) 62.0 (1.6)	1.9 (0.6 to 3.4) 1.5 (0.1 to 3.0)	**0.024** **0.035**	1.00 0.78
Velocity (cm/s)	106.3 (23.6)	124.0 (11.6)	17.7 (4.7 to 30.9)	**0.017**	0.95
Cadence (steps/min)	108 (9)	117 (6)	8.8 (3.1 to 14.6)	**0.004**	1.18
	—	—	—	—	—
CHAMPS	(*n* = 15)	(*n* = 15)	—	—	—
Duration of all physical activity (hours)	11.2 (6.0)	13.6 (5.5)	2.4 (−1.9 to 6.7)	0.268	0.42
Duration of MVPA (hours)	2.6 (2.5)	5.2 (2.7)	2.6 (0.6 to 4.5)	**0.011**	1.00
Frequency of all physical activity (bouts)	20.4 (11.1)	19.6 (8.4)	0.8 (−6.6 to 8.2)	0.825	0.08
Frequency of MVPA (bouts)	5.3 (4.8)	7.5 (6.3)	2.1 (−2.1 to 6.3)	0.305	0.39
	—	—	—	—	—
Knee-extension strength	(*n* = 9)	(*n* = 14)	—	—	—
Left (TQ/BW)	166.7 (45.2)	173.1 (56.0)	6.4 (−38.0 to 50.7)	0.768	0.12
Right (TQ/BW)	165.1 (42.8)	164.7 (46.2)	0.4 (−39.2 to 40.1)	0.982	0.01

Data are mean (SD) unless stated.

CoP: Centre of pressure; AP: Anterior-posterior; ML: Mediolateral; %GC: Percentage of gait cycle; MVPA moderate/vigorous physical activity; TQ/BW: peak torque/body weight.

Effect size = Mean between group difference/Pooled standard deviation.

#### Kistler force plate

Eleven participants in the lung cancer group and 13 in the control group completed the lab-based balance assessment. Incompletion of the test was due to technical faults with the equipment, including inability to connect to the computer and the force plate not registering the participants’ weight. No significant differences were seen in bipedal stances between groups (*p* > 0.05) (Table 2).

#### GAITRite

Step time, cycle time, heel-to-heel base of support and stance time were significantly higher among the lung cancer group (*p*<0.05). Swing time, velocity and cadence were all significantly higher among healthy controls (*p*<0.05) (Table 2).

#### Biodex

Nine participants in the lung cancer group and 14 healthy controls completed the knee-extension strength assessment. One participant in the lung cancer group declined to complete this assessment due to weakness, whilst others (*n* = 6) did not complete due to technical issues with the equipment. These issues included inability to calibrate the equipment due to updates required to the hardware, and issues with the computer not booting correctly. No significant differences were seen for either leg between groups (*p* > 0.05) (Table 2).

#### CHAMPS

Healthy controls took part in significantly longer MVPA per week compared to the lung cancer group, with a mean difference (95% CI) of 2.6 h (0.6 to 4.5 h) (*p* = 0.011). No difference was seen between groups for all PA (*p* > 0.05) (Table 2).

#### Associations between balance, strength and physical activity levels

Moderate and weak associations existed between total BESTest score and duration (r = 0.522, *p =* 0.003) and frequency (r = 0.384, *p =* 0.036) of MVPA for all participants (*n* = 30). High and moderate correlations existed between total BESTest score and knee-extension strength in healthy controls (*n* = 15) (Left: r = 0.711, *p* = 0.004; Right: r = 0.558, *p* = 0.038). No correlations were found in those with lung cancer (*n* = 15) (Table 3).

**Table 3.** Correlation (Pearson’s r and (*p* value)) between BESTest total score and: 1) knee-extension strength and 2) physical activity levels.

<!--Col Count:4-- >	Post-surgery	Control	Combined
Knee-extension strength	(*n* = 9)	(*n* = 14)	(*n* = 23)
Left	0.215 (0.578)	0.711 **(0.004)**	0.356 (0.095)
Right	0.018 (0.963)	0.558 **(0.038)**	0.187 (0.393)
CHAMPS	(*n* = 15)	(*n* = 15)	(*n* = 30)
Duration (all)	0.351 (0.199)	0.053 (0.851)	0.323 (0.082)
Duration (MVPA)	0.425 (0.115)	0.274 (0.324)	0.522 **(0.003)**
Frequency (all)	0.498 (0.059)	0.360 (0.188)	0.329 (0.076)
Frequency (MVPA)	0.336 (0.221)	0.505 (0.055)	0.384 **(0.036)**

CHAMPS: Community Healthy Activities Model Program for Seniors; MVPA: moderate/vigorous physical activity.

### Qualitative findings

#### Study population

13 individuals with lung cancer completed the two-part interview (nine male (69%), with a mean (SD) age of 68 (7) years, BMI of 27.8 (5.6) and 28.3 (23.5) pack years). Seven participants were diagnosed with a stage I cancer (Ia = 5, Ib = 2), three with a stage II cancer (IIa = 2, IIb = 1), and three with a stage III cancer (IIIa = 3). The mean (SD) time between surgery and interviews was 47.9 days (18.9 days). Following interviews with 10 participants, data saturation had been reached and was confirmed in a further three interviews.

Three themes were identified: 1) Symptoms affect daily activities; describing the influence of pain, dyspnoea and fatigue on recovery; 2) Functional assessments alter perceptions of balance ability; describing the influence of performing the assessments on peoples’ perceptions of balance ability, the tasks they found most challenging and how balance performance relates to everyday activities; and 3) Open to a supervised intervention; describing participants’ opinions on the content and delivery of a potential intervention to improve balance, gait and functional status.

Most individuals described shock at receiving a lung cancer diagnosis, positive views about the surgery and the speed at which it was performed, and discontentment about the lack of support received following hospital discharge. These findings are well described in the literature so were largely bracketed; however, they did contextualise peoples’ perceptions on functional status and support needs.

#### Symptoms affect daily activities

Most individuals described experiencing pain following surgery *“Very painful, if I didn’t have to I wouldn’t go through it again…painful and quite debilitating”* (patient ID 07), while a minority reported no pain *“I feel a bit of fraud really because I’ve never had any symptoms”* (patient ID 01). This was accompanied by intense dyspnoea and crippling fatigue *“Drastically short of breath”* (patient ID 02), *“I get more tired than I did before”* (patient ID 08). Some individuals described feeling unsteady *“if you see me first thing in the morning going for a walk down the road, you might think I’d been drinking”* (patient ID 01). Most participants portrayed difficulties in carrying out daily activities post-surgery, leading to fear and diminished self-confidence *“I’m quite nervous about going out... I wouldn’t dare go out by myself”* (patient ID 11), potentially leading to activity avoidance. Narratives portrayed a reliance on others, resulting in a loss of independence and feelings of vulnerability “*I could get out of bed on a morning and open one curtain then I had to sit back on the bed. I just couldn’t do it, completely and utterly breathless”* (patient ID 02).

#### Functional assessments alter perceptions of balance

Narratives described standing on one leg or on uneven surfaces, particularly with eyes closed, as challenging. Yet, individuals felt these tasks to be arbitrary and not related to everyday life *“It’s not something that is in normal life. You know, if you go to ASDA [supermarket] you don’t stand on one leg or with your eyes closed”* (patient ID 02), meaning any impact of balance performance on confidence and behaviour was limited. Some participants believed their balance was worse following the assessments “*My balance is worse than I thought it was going to be*” (patient ID 07), whilst others’ opinions did not alter. One patient even described their balance as “*better than I thought it was”* (patient ID 10).

#### Open to a supervised intervention

Most participants expressed openness and enthusiasm for a rehabilitation intervention following surgery to address balance and gait impairments, and to improve functional status *“If I had been told that there is a programme designed around your particular condition. Then yes I would have willingly taken it on board”* (patient ID 03). Only one participant expressed a preference for home-based rehabilitation, finding it a more flexible option “…*you don’t know when you’re going to get that half an hour… And if I want to do it while I’m still in me pyjamas I can do it still in my pyjamas”* (patient ID 10). Most participants wished an intervention to be supervised by a healthcare professional, namely a physiotherapist, citing concerns about safety when exercising alone *“You would need somebody there to actually manage a programme… that would be another thing for safety, if you were given a programme by somebody and they were there to watch you go through it, and see how you were”* (patient ID 09).

Participants had mixed preferences for the frequency and duration of a programme. Commonly, they suggested undertaking the intervention once per week for 1 hour; however, this ranged from 15 min to 2 hours, with exercise frequency ranging from daily to monthly. Seven participants identified group-based programmes to be of greater interest *“I would do group-based and then you can get people to encourage each other. You know, and have a bit of a laugh with each other, falling over you know… I mean they’re all going to be lung cancer patients. So they might just gel together if you had it as a group”* (patient ID 06). Four participants conveyed a preference to complete an intervention alone, as they felt they did not need additional support or encouragement, whilst two had no preference. Of all participants, only one was opposed to receiving any intervention as they did not perceive any impairments.

## Discussion

This is the first study to investigate balance and gait in individuals with lung cancer post-thoracic surgery. Clinically significant between group differences were found for the BESTest (the primary outcome). Dynamic balance and gait were impaired, with time spent participating in MVPA less than healthy age-matched adults. This is likely due to symptoms of pain, dyspnoea and fatigue experienced even 3 months post-surgery. Yet, participants did not believe balance to be impaired, nor did they perceive this to have any impact on daily activities. However, the offer of rehabilitation following surgery was welcomed, perhaps to compensate for a sense of abandonment following hospital discharge and to address any lingering symptoms.

Those who had recently undergone thoracic surgery for a diagnosis of lung cancer performed worse on the BESTest than healthy age-matched adults. Scores are consistent with other chronic disease populations known to have poor balance (68% lung cancer vs. 66–71% in COPD, multiple sclerosis and Parkinson’s disease).^15,36–39^ The assessments that participants found most difficult included one-legged stance and balancing with eyes closed, perhaps indicating a reliance on vision, which was not controlled for in the current study but should be considered in future research. Performance on bipedal static balance assessments was similar between groups. This is important because dynamic balance is more akin to real life, indicating that it might be co-ordination or the ability to dual task which poses difficulties. Inability to dual task has been shown to affect gait among cancer survivors.^40^ Despite this, most participants did not believe balance to be impaired post-surgery, potentially due to not seeing their performance on these assessments as an indicator of falls in everyday life.

A cautious gait potentially indicates a fear of falling and might explain why MVPA is low compared to healthy controls. Walking at a speed of 1.0 m/s to 1.3 m/s is reported as normal and is associated with a lower risk of falling.^41^ People with lung cancer in this study walked at 1.06 m/s, perhaps to try and avoid falling. Falls history and fear of falling were not assessed within this study; however, they would be important to consider in future research.

Quadriceps strength was similar between groups, despite participants reporting feeling weaker since surgery and being unable to carry out their usual daily activities. These findings are similar to people with COPD (peak torque normalised by body weight: 166%).^42^ In a previous study, quadriceps strength was assessed using a handheld dynamometer in people with lung cancer pre-treatment, 10 weeks after diagnosis, and six months after diagnosis. Quadriceps strength was shown to be impaired at the time of diagnosis compared to healthy controls (18.8 kg v 23.7 kg), and further declined over a period of 6 months (14.4 kg).^43^ Yet, strength in the current study was not associated with balance, despite this having been found in people with COPD.^44^

Individuals with lung cancer displayed similar mild to moderate PA levels to healthy controls, indicating they are maintaining PA to carry out essential daily activities (e.g. washing and dressing) but are avoiding strenuous activities, as shown by a lower MVPA. This may also mean that individuals are not challenging themselves in real world situations which could be why they did not perceive their balance to be impaired. Avoiding such activities might result in an increased fear of falling, due to not knowing if they could maintain balance when performing MVPA or it may be why they avoid these activities. We recruited family members of individuals with lung cancer, including spouses, so lifestyle factors are likely matched, which might be why no difference was found between groups in mild to moderate PA or strength. These findings contradict previous literature, which found that individuals with lung cancer at the time of diagnosis engaged in significantly less physical activity than healthy controls, following completion of the Physical Activity Scale for the Elderly (83.9 v 161.9).^43^ However, quadriceps strength in this sample was similar (169%) to other healthy populations (174%).^15^

There was no association between dynamic balance and strength or PA levels for the lung cancer group, yet balance was significantly associated with strength for healthy controls. Strength, therefore, is unlikely to be the reason why balance is impaired in this population, rather it is likely due to other factors such as side effects of surgery (e.g. increased pain and altered sense of gravity due to anatomical changes). However, the sample size is small, and we should be cautious interpreting these results. The mechanisms underpinning balance impairment in this population requires further exploration. Implementing an intervention to improve balance and gait in this population might be difficult, as most did not perceive these to be impaired. However, participants stated that if an intervention were in place, balance and cardio exercises would be most beneficial, supporting observations from the physical assessments. Although most post-surgery symptoms will resolve with time, some may be long-lasting, such as dyspnoea due to reduced lung capacity, and an altered COG due to anatomical changes. Therefore, an intervention to target balance may be required in the long-term.

### Limitations

We were unable to recruit the desired sample size (*n* = 17) in each group due to difficulties with recruitment and the onset of the COVID-19 pandemic. This meant the study was slightly underpowered; however, there was 74% and 92% power (*p* = 0.05) to detect the lower and upper MCID of 13 and 17 points.^16^ Also, due to technical difficulties with some equipment, not all participants completed every assessment, which impacted the sample size and reduces the confidence we can have in the findings. No measures were completed pre-surgery. It is difficult to obtain pre-measures in thoracic surgery settings due to the short period of time between diagnosis and surgery, which can be as little as 2 days. The symptoms that participants were experiencing at the time of the assessments were not collected, which may have provided greater understanding of the effects of surgery, such as pain and dyspnoea. These could have been assessed using the Brief Pain Inventory and MRC dyspnoea scale, respectively. There was a significant correlation between BESTest total score and duration of MVPA when the data for both groups were combined. This finding must be interpreted with caution due to the variance of the MVPA results, meaning the combined correlation was greater than that of each group.

There was up to 3 months between surgery and interviews; therefore, some important information about the surgery and associated symptoms may be forgotten.^45–47^ Future work should also look to assess falls history and future falls. This could be done by using a self-reported falls diary prior to and following surgery. Fear of falling among participants is also important to consider, as it has been linked to a reduction in physical activity levels and other risk factors associated with falls (e.g. slow gait velocity and poor balance).^48^ It would also be beneficial to explore the long-term impact of thoracic surgery for the treatment of lung cancer and whether any impairments in balance are long-lasting or improve with time due to the process of natural recovery. Interventions to improve balance and gait need to be endorsed by the cardiothoracic surgeons and the clinical team to aid engagement. Clinicians (including nurses and physios) could also be involved in the design and delivery of an intervention.

## Conclusion

Dynamic balance and gait are impaired in people with lung cancer following thoracic surgery, compared to healthy adults of the same age. These impairments could not be explained by quadriceps strength or PA levels. Participants did not believe poor balance impacted their ability to carry out everyday activities. Yet, a supervised group-based intervention to target balance and gait would be welcomed.

## Supplemental Material

sj-pdf-1-crd-10.1177_14799731211052299 – Supplemental Material for Investigating balance, gait, and physical function in people who have undergone thoracic surgery for a diagnosis of lung cancer: A mixed-methods study Click here for additional data file. Supplemental Material, sj-pdf-1-crd-10.1177_14799731211052299 for Investigating balance, gait, and physical function in people who have undergone thoracic surgery for a diagnosis of lung cancer: A mixed-methods study by Daniel Tough, Joel Dunning, Jonathan Robinson, John Dixon, Jonathan Ferguson, Ian Paul and Samantha L Harrison in Chronic Respiratory Disease

## References

[1] Cancer Research UK . Cancer Mortality for Common Cancers, 2019. https://www.cancerresearchuk.org/health-professional/cancer-statistics/mortality/common-cancers-compared#heading-Zero (accessed 27 February 2021).

[2] Blichfeldt-Eckhardt MR Andersen C Ørding H , et al. From acute to chronic pain after thoracic surgery: the significance of different components of the acute pain response. J Pain Res 2018; 11: 1541–1548. 30147358 10.2147/JPR.S161303 PMC6101742

[3] Jastrzębski D Żebrowska A Rutkowski S , et al. Pulmonary Rehabilitation with a Stabilometric Platform after Thoracic Surgery: A Preliminary Report. J Hum Kinetics 2018; 65: 79–87. 10.2478/hukin-2018-0044 PMC6341951 30687421

[4] Kocjan J Gzik-Zroska B Nowakowska K , et al. Impact of diaphragm function parameters on balance maintenance. PLoS ONE 2018; 13(2): e0208697. 30592726 10.1371/journal.pone.0208697 PMC6310257

[5] Huang X Zhou W Zhang Y . Features of fatigue in patients with early-stage non-small cell lung cancer. J Res Med Sci 2015; 20(3): 268–272. 26109974 PMC4468232

[6] Williams AC Grant M Tiep B , et al. Dyspnea management in early stage lung cancer: a palliative perspective. J Hosp Palliat Care 2012; 14(5). 10.1097/NJH.0b013e31825e4250 PMC3776584 24058283

[7] Schulte T Schniewind B Dohrmann P , et al. The extent of lung parenchyma resection significantly impacts long-term quality of life in patients with non-small cell lung cancer. Chest 2009; 135(2): 322–329. 18689576 10.1378/chest.08-1114

[8] Synnott E Baker K . The effectiveness of vestibular rehabilitation on balance related impairments among multiple sclerosis patients: a systematic review. J Mult Scler 2020; 7(1): 1–8.

[9] Huang MH Blackwood J Godoshian M , et al. Prevalence of self-reported falls, balance or walking problems in older cancer survivors from surveillance, epidemiology and end results—medicare health outcomes survey. J Geriatr Oncol 2017; 8(4): 255–261. 28602712 10.1016/j.jgo.2017.05.008

[10] Lee AL Zabjek K Goldstein RS , et al. Systematic review of postural assessment in individuals with obstructive respiratory conditions: measurement and clinical associations. J Cardiopulmonary Rehabil Prev 2017; 37(2): 90–102. 27676462 10.1097/HCR.0000000000000207

[11] Overcash J Beckstead J . Predicting falls in older patients using components of a comprehensive geriatric assessment. Clin J Oncol Nurs 2008; 12(6): 941–949. 19064388 10.1188/08.CJON.941-949

[12] Harun A Li C Bridges JFP , et al. Understanding the experience of age-related vestibular loss in older individuals: a qualitative study. Patient 2016; 9(4): 303–309. 26739817 10.1007/s40271-015-0156-6 PMC4925240

[13] Coulman KD MacKichan F Blazeby JM , et al. Patient experiences of outcomes of bariatric surgery: a systematic review and qualitative synthesis. Obes Rev 2017; 18(5): 547–559. 28273694 10.1111/obr.12518 PMC5709707

[14] Harrison SL Watson P Milburn C , et al. Perceptions of early discharge following lung surgery: I’m a patient “get me out of here. J Hosp Manag Health Pol 2019; 3.

[15] Beauchamp MK Sibley KM Lakhani B , et al. Impairments in systems underlying control of balance in COPD. Chest 2012; 141(6): 1496–1503. 22116798 10.1378/chest.11-1708

[16] Beauchamp MK Harrison SL Goldstein RS , et al. Interpretability of change scores in measures of balance in people with COPD. Chest 2016; 149(3): 696–703. 26203790 10.1378/chest.15-0717

[17] Horak FB Wrisley DM Frank J . The Balance Evaluation Systems Test (BESTest) to differentiate balance deficits. Phys Ther 2009; 89(5): 484–498. 19329772 10.2522/ptj.20080071 PMC2676433

[18] Jácome C Cruz J Oliveira A , et al. Validity, reliability, and ability to identify fall status of the berg balance scale, BESTest, Mini-BESTest, and Brief-BESTest in Patients With COPD. Phys Ther 2016; 96(11): 1807–1815. 27081201 10.2522/ptj.20150391

[19] García-Massó X Pellicer-Chenoll M Gonzalez LM , et al. The Difficulty of the Postural Control Task Affects Multi-Muscle Control During Quiet Standing. Exp Brain Res 2016; 234(7): 1977–1986. 26942928 10.1007/s00221-016-4602-z PMC4893067

[20] Meshkati Z Namazizadeh M Salavati M , et al. Reliability of Force-Platform Measures of Postural Sway and Expertise-Related Differences. J Sport Rehabil 2011; 20(4): 442–456. 22012498 10.1123/jsr.20.4.442

[21] Moghadam M Ashayeri H Salavati M , et al. Reliability of center of pressure measures of postural stability in healthy older adults: Effects of postural task difficulty and cognitive load. Gait and Posture 2011; 33(4): 651–655. 21458272 10.1016/j.gaitpost.2011.02.016

[22] Bauer C Gröger I Rupprecht R , et al. Reliability analysis of time series force plate data of community dwelling older adults. Arch Gerontol Geriatr 2010; 51(3): e100–105. 20153904 10.1016/j.archger.2010.01.009

[23] Pinsault N Vuillerme N . Test–retest reliability of centre of foot pressure measures to assess postural control during unperturbed stance. Med Eng Phys 2008; 31(2): 276–286. 18835738 10.1016/j.medengphy.2008.08.003

[24] Lim Y Ko P Park K , et al. Quantitative Gait Analysis and Cerebrospinal Fluid Tap Test for Idiopathic Normal-pressure Hydrocephalus. Scientific Rep 2019; 9(1): 16255. 10.1038/s41598-019-52448-3 PMC6838166 31700018

[25] Marshall TF Zipp GP Battaglia F , et al. Chemotherapy-induced-peripheral neuropathy, gait and fall risk in older adults following cancer treatment. J Cancer Res Pract 2017; 4(4): 134–138.

[26] Khan F Anjamparuthikal H Chevidikunnan MF . The comparison between isokinetic knee muscles strength in the ipsilateral and contralateral limbs and correlating with function of patients with stroke. J Neurosciences Rural Pract 2019; 10(4): 683–689. 10.1055/s-0039-1700612 PMC6906114 31831990

[27] Bohannon RW Bubela DJ Wang Y , et al. Adequacy of belt-stabilized testing of knee extension strength. J Strength Conditioning Res 2011; 25(7): 1963–1967. 10.1519/JSC.0b013e3181e4f5ce PMC3116990 21399535

[28] Kilgour RD Vigano A Trutschnigg B , et al. Cancer-related fatigue: the impact of skeletal muscle mass and strength in patients with advanced cancer. J Cachexia Sarcopenia Muscle 2010; 1(2): 177–185. 21475694 10.1007/s13539-010-0016-0 PMC3060645

[29] de Vasconcelos R Bevilaqua-Grossi D Shimano AC , et al. Reliability and validity of a modified isometric dynamometer in the assessment of muscular performance in individuals with anterior cruciate ligament reconstruction. Revista Brasileira de Ortopedia 2009; 44(3): 214–224. 27004175 10.1016/S2255-4971(15)30071-9 PMC4783672

[30] Stewart AL Mills KM King AC , et al. CHAMPS Physical Activity Questionnaire for Older Adults: outcomes for interventions. Epidemiology 2001; 33(7): 1126–1141. 10.1097/00005768-200107000-00010 11445760

[31] Hekler EB Buman MP Haskell WL , et al. Reliability and Validity of CHAMPS Self-Reported Sedentary-To-Vigorous Intensity Physical Activity in Older Adults. J Phys Activity Health 2012; 9(2): 225–236. 10.1123/jpah.9.2.225 PMC4733646 22368222

[32] Giles K Marshall A . Repeatability and accuracy of CHAMPS as a measure of physical activity in a community sample of older Australian adults. J Phys Activity Health 2009; 6(2): 221–229. 10.1123/jpah.6.2.221 19420400

[33] Chen BP Awasthi R Sweet SN , et al. Four-week prehabilitation program is sufficient to modify exercise behaviors and improve preoperative functional walking capacity in patients with colorectal cancer. Support Care Cancer 2017; 25(1): 33–40. 27539131 10.1007/s00520-016-3379-8

[34] Pinto BM Papandonatos GD Goldstein MG , et al. Home‐based physical activity intervention for colorectal cancer survivors. Psycho-Oncology 2013; 22(1): 54–64. 21905158 10.1002/pon.2047

[35] Braun V Clarke V . Using thematic analysis in psychology. Qual Res Psychol 2006; 3(2): 77–101.

[36] Beauchamp MK Janaudis-Ferreira T Parreira V , et al. A randomized controlled trial of balance training during pulmonary rehabilitation for individuals with COPD. Chest 2013; 144(6): 1803–1810. 23975185 10.1378/chest.13-1093

[37] Mitchell KD Chen H Silfies SP . Test-retest reliability, validity, and minimal detectable change of the balance evaluation systems test to assess balance in persons with multiple sclerosis. Int J MS Care 2018; 20(5): 231–237. 30374253 10.7224/1537-2073.2016-118 PMC6200120

[38] Duncan RP Leddy AL Cavanaugh JT , et al. Detecting and Predicting Balance Decline in Parkinson Disease: a Prospective Cohort Study. J Parkinsons Dis 2015; 5(1): 131–139. 25514984 10.3233/JPD-140478 PMC4843994

[39] Leddy AL Crowner BE Earhart GM . Utility of the Mini-BESTest, BESTest, and bestest sections for balance assessments in individuals with Parkinson Disease. J Neurol Phys Ther 2011; 35(2): 90–97. 21934364 10.1097/NPT.0b013e31821a620c PMC3178037

[40] Monfort S Pan X Loprinzi CL , et al. Exploring the roles of central and peripheral nervous system function in gait stability: preliminary insights from cancer survivors. Gait & Posture 2019; 71: 62–68. 31009918 10.1016/j.gaitpost.2019.04.002 PMC6594171

[41] Quach L Galica AM Jones RN , et al. The nonlinear relationship between gait speed and falls: the maintenance of balance, independent living, intellect, and zest in the elderly of Boston study. J Am Geriatr Soc 2011; 59(6): 1069–1073. 21649615 10.1111/j.1532-5415.2011.03408.x PMC3141220

[42] Butcher SJ Pikaluk BJ Chura RL , et al. Associations between isokinetic muscle strength, high-level functional performance, and physiological parameters in patients with chronic obstructive pulmonary disease. Int J Chronic Obstructive Pulm Dis 2012; 7: 537–542. 10.2147/COPD.S34170 PMC3430119 22973094

[43] Granger CL McDonald CF Irving L. , et al. Low physical activity levels and functional decline in individuals with lung cancer. Lung Cancer 2014; 83(2): 292–299. 24360323 10.1016/j.lungcan.2013.11.014

[44] Loughran KJ Atkinson G Beauchamp MK , et al. Balance impairment in individuals with COPD: a systematic review with meta-analysis. Thorax 2020; 75(7): 539–546. 32409612 10.1136/thoraxjnl-2019-213608

[45] Garcia PA Dias JMD Silva SLA , et al. Prospective monitoring and self-report of previous falls among older women at high risk of falls and fractures: a study of comparison and agreement. Braz J Phys Ther 2015; 19(3): 218–226. 26083603 10.1590/bjpt-rbf.2014.0095 PMC4518575

[46] Jones G Tabassum V Zarow GJ , et al. The inability of older adults to recall their drugs and medical conditions. Drugs and Aging 2015; 32(4): 329–336. 25829296 10.1007/s40266-015-0255-z

[47] Bherer L Erickson KI Liu-Ambrose T . A review of the effects of physical activity and exercise on cognitive and brain functions in older adults. J Aging Res 2013; 2013: 657508. 24102028 10.1155/2013/657508 PMC3786463

[48] Young WR Williams AM . How fear of falling can increase fall-risk in older adults: Applying psychological theory to practical observations. Gait and Posture 2015; 41(1): 7–12. 25278464 10.1016/j.gaitpost.2014.09.006

