# Supplementary material for: Investigating balance, gait, and physical function in people who have undergone thoracic surgery for a diagnosis of lung cancer: A mixed-methods study
Source: Chron Respir Dis. 2021 Oct 29;18:14799731211052299. doi: 10.1177/14799731211052299 (PMC8558594; doi:10.1177/14799731211052299)
Supplement: sj-pdf-1-crd-10.1177_14799731211052299 – Supplemental Material for Investigating balance, gait, and physical function in people who have undergone thoracic surgery for a diagnosis of lung cancer: A mixed-methods study [file sj-pdf-1-crd-10.1177_14799731211052299.pdf]

Supplementary material

Supplement 1 Interview Schedule

Before Testing

The participation within this interview is voluntary, and you can withdraw from the interview or not answer any questions you don't wish to. You can still partake in the study testing if you do not complete the interview.

Q. Would you like to tell me a little bit about your diagnosis and your surgery?

Q. Have you had any trips, falls or losses of balance since your surgery? If so, could you tell me about it?

(Prompt: Injury, uneven surface, wet floor)

Q. How do you feel your strength is since your surgery? In particular your leg strength?

(Prompt: feel weaker, more wobbly, unsteady, less confident)

Q. How do you feel your balance is?

Q. How does your balance affect your ability to do everyday tasks?

(Prompt: shopping, cleaning, hobbies, less walking)

Q. Do you need to ask for assistance with any walking or anything requiring you to balance? If so, what sort of tasks do you struggle with and what assistance do you need? (Prompt: Stair climbing, walking aids, sit to stand)

Q. Does your balance have an effect on your confidence?

(Prompt: Going to shops, walking the streets, doing new tasks)

After Testing

Q. How did you feel during the testing?

(Prompts: did you enjoy it, did you feel worried during any of the tests?)

Q. What test(s) did you find to be most challenging, and why?

(Prompt: BESTest/force plate balance/walking/leg strength)

Q. Following these tests, has your opinion of your balance changed? If so, in what way?

Q. Had a rehabilitation method for improving your balance been available for you following surgery, what would you expect this to look like?

(Prompt: What exercises, frequency, duration, who would you have wanted to deliver it)

Q. Where would you prefer to receive a rehabilitation program, and why?

(Prompt: Home, hospital, gym)

Q. Had a rehabilitation program been in place, would you rather this be carried out alone or with a group of others under supervision?

(Prompt: Alone at home, group based at clinic/gym/hospital with an instructor)

Q. Would a standard rehabilitation program or a technology based program be most appealing, and why?

(Prompt: Nintendo Wii/Xbox Kinect games, mobile app, list of standard physio exercises)

Supplement 2. Example of quantitative and qualitative triangulation process

Physical assessment	Interview quotes	Triangulation
Total BESTest score (%)<br>Post-surgery: 68.1<br>Controls: 88.0<br>(*p*=**0.003**)	*"I haven't noticed any significant change in my balance"* (patient ID 03)<br><br>*"It's not something that is in normal life. You know, if you go to ASDA you don't stand on one leg or with your eyes closed"* (patient ID 02)	The post-surgery group had significantly worse balance than the control group, although most participants did not believe that their balance was impaired. Participants did not see their performance on the tasks involved in the physical assessments as an indicator of falls in everyday life, rather they disassociated the importance of this.
Knee-extension strength (TQ/BW)<br>Left<br>Post-surgery: 166.7<br>Control: 175.1<br>(*p*=0.768)<br>Right<br>Post-surgery: 165.1<br>Control: 164.7<br>(*p*=0.982)	*"I don't feel terribly strong at the moment"* (patient ID 01)<br><br>*"I can remember picking up the vacuum cleaner initially and it was so, so heavy. And now I can just pick it up"* (patient ID 05)	Quadriceps strength was unaffected in the post-surgery group, despite participants portraying feeling weaker since surgery and being unable to carry out their usual daily activities. Strength may, therefore, not be the best indicator of physical function among this population, due to contradicting findings between the physical assessments and participant interviews.
Physical activity levels (Duration of MVPA (hours))<br>Post-surgery: 2.6<br>Control: 5.2<br>(*p*=**0.011**)	*"I think the only main thing is mentally I think I can do more that I can do physically. I get more tired than I did before"* (patient ID 08)<br><br>*"I'm a bit nervous, quite nervous about going out... I wouldn't dare go out by myself"* (patient ID 11)	There was no significant difference in light physical activity between groups; however, the post-surgery group spent significantly less time doing MVPA. This indicates the post-surgery group are avoiding too many strenuous activities, potentially due to their perceived decrease in strength or pain, which was expressed by participants describing being unable to carry out difficult everyday tasks following surgery. The findings from the interviews compliment that of the physical assessments, potentially giving an explanation as to why lower MVPA was seen in this group.

TQ/BW: peak torque/body weight; MVPA: moderate/vigorous physical activity
